# Specialization on pollen or nectar in bumblebee foragers is not associated with ovary size, lipid reserves or sensory tuning

**DOI:** 10.7717/peerj.2599

**Published:** 2016-10-27

**Authors:** Adam R. Smith, Peter Graystock, William O.H. Hughes

**Affiliations:** 1 Department of Biological Sciences, George Washington University, Washington, DC, United States; 2 Department of Entomology, University of California, Riverside, Riverside, CA, United States; 3 School of Life Sciences, University of Sussex, Brighton, United Kingdom

**Keywords:** Reproductive ground plan hypothesis, Foraging specialization, Division of labor

## Abstract

Foraging specialization allows social insects to more efficiently exploit resources in their environment. Recent research on honeybees suggests that specialization on pollen or nectar among foragers is linked to reproductive physiology and sensory tuning (the Reproductive Ground-Plan Hypothesis; RGPH). However, our understanding of the underlying physiological relationships in non-*Apis* bees is still limited. Here we show that the bumblebee *Bombus terrestris* has specialist pollen and nectar foragers, and test whether foraging specialization in *B. terrestris* is linked to reproductive physiology, measured as ovarian activation. We show that neither ovary size, sensory sensitivity, measured through proboscis extension response (PER), or whole-body lipid stores differed between pollen foragers, nectar foragers, or generalist foragers. Body size also did not differ between any of these three forager groups. Non-foragers had significantly larger ovaries than foragers. This suggests that potentially reproductive individuals avoid foraging.

## Introduction

Eusociality is characterized by the division of colonies into a reproductive queen and non-reproductive workers. Among the worker caste, there may be further task specialization, especially in large-colony species with derived social organization. For instance, honeybee (*Apis mellifera*) foragers may specialize on collecting nectar or pollen (Free, 1960). Understanding the mechanisms underlying such specialization can suggest how worker task specialization and its attendant social coordination evolved. The reproductive groundplan hypothesis (RGPH) proposes a mechanism for the evolution of pollen/nectar foraging preference in bees in which reproductive physiology is linked to foraging specialization (Amdam et al., 2004; Amdam et al., 2006; Page et al., 2006; Page, Rueppell & Amdam, 2012; Page & Amdam, 2007; Roth et al., 2014).

Studies of honeybees show that individual pollen foraging specialists have more developed ovaries with more ovarioles than nectar foragers (although both groups are typically non-reproductive; Amdam et al., 2004; Amdam et al., 2006; Page & Amdam, 2007; Page, Rueppell & Amdam, 2012). Pollen foragers also have more sensitive sensory tuning; it takes a lower concentration of sugar touched to the antennae to provoke a pollen specialist to extend her proboscis than a nectar forager (the “Proboscis Extension Response,” PER; Page et al., 2006). This sensory sensitivity is not limited to sugar, lower concentrations of pollen will also elicit PER from pollen foragers than from nectar foragers (Page et al., 2006). This link between reproductive physiology and foraging behavior reflects the hypothesized reproductive groundplan of ancestral solitary bees; when reproductive they forage for pollen as a source of protein, but when non-reproductive, only nectar as a carbohydrate for self-maintenance. In the eusocial honeybee, these ancestral regulatory mechanisms linking reproductive development with sensory tuning have been repurposed for foraging specialization rather than reproduction (Amdam et al., 2004; Amdam et al., 2006; Page et al., 2006; Page, Rueppell & Amdam, 2012; Page & Amdam, 2007). Roth et al. (2014) label this the Reproductive Ground Plan-forager hypothesis to distinguish the focus on honeybee foraging specialization from the broader original RGPH and other related hypotheses focusing on the evolutionary origins of eusociality from solitary ancestors (West-Eberhard, 1989; West-Eberhard, 1996; Amdam et al., 2004; Hunt, 2007). Here we use RGPH to be consistent with earlier authors, although our focus is on foraging specialization.

All RGPH studies of pollen/nectar foraging specialization to date have been on *A. mellifera* or other species or strains of *Apis* (“anarchic” *A. mellifera* (Oldroyd & Beekman, 2008), *A. cerana* (Rueppell, Hunggims & Tingek, 2008; Tan et al., 2015), *A. m. capensis* (Roth et al., 2014)). Bumblebees (*Bombus* spp.) also have pollen and nectar specialist foragers (Verhaeghe et al., 1999; O’Donnell, Reichardt & Foster, 2000; Hagbery & Nieh, 2012; Konzmann & Lunau, 2014). In this study, we test whether pollen and nectar foraging specialization in bumblebees is linked to reproductive physiology as in honeybees. We test the prediction that pollen specialist foragers of the bumblebee *Bombus terrestris* have larger ovaries and more sensitive sensory tuning than nectar specialist foragers. In honeybees variation in ovarian development is seen in ovariole number, which is variable among individuals but fixed during adult development (Amdam et al., 2004; Amdam et al., 2006; Page & Amdam, 2007; Page, Rueppell & Amdam, 2012), However in non-parasitic *Bombus,* including *B. terrestris*, ovariole number is always eight (Cumber, 1949; Iwata, 1955; Amsalem et al., 2015), so we instead measure ovary activation, the width of the largest oocyte, as a measure of reproductive development. This is a dynamic measure that can change during adult life (e.g., Van Honk & Hogeweg, 1981). Bumblebees are not the sister taxon to *Apis,* although they are closely related in the subfamily Apinae (Danforth et al., 2013). Comparing and contrasting foraging specialization in the two groups may reveal the extent to which each evolved pollen and nectar specialization using similar mechanisms or via novel pathways.

Another hypothesis linking ovary activation to foraging (although unrelated to foraging specialization) is the ‘Reproductive Conflict and Work’ hypothesis; potentially reproductive workers may forgo foraging altogether to save themselves for reproduction (Schmid-Hempel, 1990; Roth et al., 2014). In the cape honeybee (*A. mellifera capensis*), workers with activated ovaries forage less, not more for pollen (Roth et al., 2014). In bumblebees, Jandt & Dornhaus (2011) found that non-foraging *B. impatiens* were most likely to enlarge their ovaries following queen removal. This hypothesis predicts that non-foragers will have larger ovaries than foragers, whatever their specialization, and is not mutually exclusive with the prediction that ovary differences drive pollen/nectar specialization among foragers.

Bumblebees have much smaller colonies than honeybees (low hundreds of individuals in bumblebees vs. thousands in honeybees) with less complicated social organization (Goulson, 2010). Pollen or nectar specialization among *B. terrestris* foragers has never been quantified across whole colonies, but specialization of at least some individuals has been previously noted (Verhaeghe et al., 1999; Konzmann & Lunau, 2014). However, specialization, defined as individuals that forage on pollen or nectar more frequently than expected relative to the colony as a whole, has been shown in *B. bifarius*(O’Donnell, Reichardt & Foster, 2000) and *B. impatiens* (Hagbery & Nieh, 2012). Foraging specialization in bumblebees is not absolute; all bees forage for at least some nectar, and most at least sometimes forage for pollen (Verhaeghe et al., 1999; O’Donnell, Reichardt & Foster, 2000; Hagbery & Nieh, 2012; Konzmann & Lunau, 2014). Hagbery & Nieh (2012) showed that *B. impatiens* specialists exhibit lifelong foraging preferences, such that foraging choice during a bee’s first day out of the nest strongly predicted lifelong foraging specialization. While this behavior was consistent, it was not rigid: bees adjusted their behavior to compensate for removal of other foragers, but then resumed their original behavior when the removed foragers were returned to the nest (Hagbery & Nieh, 2012).

In this study we recorded the foraging decisions of individual bees in four lab colonies of *B. terrestris* given access to both sugar water (artificial nectar) and pollen in separate feeders to quantify foraging specialization. We then tested the PER of a subset of bees at four different sugar concentrations from two of the colonies. All bees in all colonies were then collected for dissection to measure ovary size, and ether extraction of lipids to measure individual energetic reserves. These data allowed us to answer the questions: (1) do *B. terrestris* foragers demonstrate foraging specialization on pollen or nectar; (2) are foragers with relatively larger ovaries more likely to forage for pollen than nectar; (3) are individuals with greater sensory sensitivity more likely to forage for pollen than nectar; (4) do fat reserves correlate with foraging specialization? This is the first study to directly test for pollen or nectar specialization in *B. terrestris*, and the first to test for an effect of reproductive physiology on foraging specialization in a species other than *Apis* spp. honeybees.

## Methods

Bumblebee colonies were obtained from Koppert Biological Systems and found to be free from infection by the six parasites found to commonly infect bumblebee workers in commercially produced colonies as in Graystock et al. (2013). For each colony, the nest box containing the hive as shipped from Koppert was placed within a plastic flight cage (79.5 cm × 39.5 cm × 25 cm; length × width × height). The colony was placed at one end of the flight cage, and at the other end the bees were provided with a petri dish filled with 30% v/w sucrose in water in one corner and a Petri dish with pollen in the other corner following Hagbery & Nieh (2012). Pollen was obtained from Koppert and had originally been collected by honeybees. All pollen was from the same batch, and was ground before presentation to the bumblebees. Thus, the bees leaving the hive had access to a nectar feeder on one side of the far end of their cage and a pollen feeder on the other. All bees except the queens (distinguishable due to their size) were marked with numbered colored disks glued on their thorax with Krazy Glue gel, a polyacrylamide adhesive. Bees were removed from the colony with foreceps and refrigerated to enable marking. Colony size at collection ranged from 89 to 236 individual females (Table 1). All colonies were kept in temperature controlled rooms set at 28 °C. All colonies were queenright and without males.

**Table 1:** The foraging category of all bees included in the study.

	Hive 4	Hive 6	Hive 8	Hive 9	Total
Nectar	32	7	21	11	71
Pollen	29	7	16	16	68
No foraging	8	47	36	14	105
Generalist	46	8	86	28	168
1–14 trips	40	20	77	32	169
Total	155	89	236	101	581

Hive entrances were opened and size excluder removed, allowing all bees access to the feeders for 1 h per day, following Hagbery & Nieh (2012). Each feeder was observed for alternating 30 s intervals during this hour. All individuals seen actively gathering pollen or drinking nectar (proboscis extended) during that scan were counted. Individuals that left and returned within the same interval were only counted once, and an individual could be counted in consecutive scans without returning to the nest if they were still (or again) gathering pollen or imbibing nectar. Colonies 4 and 9 were observed for 8 h, Colony 6 for 5 h, and Colony 8 for 4 h. Observations for each colony took place on consecutive days, after which bees were either collected into ethanol or placed in PER harnesses. All bees were collected directly from the nest box after foraging had ceased. All observations took place between 4-22 August 2012.

***PER:*** Bees were removed from the colony placed in PER harnesses and were then given access to cotton wool soaked in 60% sucrose for 30 min to feed until satiation. Bees were then left for ∼4 h following Graystock et al. (2013) and Graystock et al. (2016). Tests were conducted under dim red light to avoid visual responses by the bees. Because few bumblebees respond to low concentrations of sucrose without repeated trials (Laloi et al., 1999; Riveros & Gronenberg, 2009) and we were interested in unlearned basal responsiveness, we used higher concentrations of sucrose than previous work on honeybees. Bees were presented with sucrose solutions of 60, 70, 80, and 90% v/w, presented in increasing concentration with 20 min between trials. A small ball of cotton wool soaked in sucrose solution was touched to the tip of the bee’s left antenna. A positive response involved the bee extending its proboscis. Each bee received a PER score that was the sum of its positive responses (0–4). PER tests were conducted on bees from colony 8 (*n* = 78) and colony 6 (*n* = 61).

***Ovary measurement:*** Upon collection, either directly from the colony or at the conclusion of the PER trials, bees were placed into 95% ethanol and stored at −20 °C. Dissections took place in 95% ethanol at 10× magnification. The width of the largest oocyte of either ovary was measured using an ocular micrometer (*n* = 98, 81, 217, 87 for colonies 4, 6, 8, 9, respectively) following standard methods (e.g., Martins & Serrã, 2004; Biani & Wcislo, 2007; Cini, Meconcelli & Cervo, 2013).

***Lipid extraction:*** after dissection, the complete bee carcass was placed into a pre-weighed vial. The vials were left open in a drying oven to evaporate the ethanol and then weighed. The bees were then soaked in ether, dried, and re-weighed (Ellers, 1995; Brown, Loosli & Schmid-Hempel, 2000; Strohm, 2000; O’Neill et al., 2011; O’Neill, Delphia & Pitts-Singer, 2015; Graystock et al., 2016). The difference between the two dry weights represents the amount of lipids present in each bee (*n* = 132, 80, 216, 85, for colonies 4, 6, 8, 9, respectively) and is reported as a percentage of total body weight to standardize for intra-individual size variation.

***Body size:*** the right rear leg of each bee was removed and affixed to a microscope slide with transparent tape. The slide was photographed at 10×, and the length of the femur measured from the photograph using Image J (NIH) as a measure of body size (*n* = 139, 89, 217, 88 for colonies 4, 6, 8, 9 respectively).

### Statistical analyses

Foraging specialists were determined by using a binomial test following O’Donnell, Reichardt & Foster (2000). Those bees that significantly (exact binomial test two-tailed *p* < 0.05) deviated from expected values (computed from colony means of total number of nectar and pollen observations) were categorized as specialists; other foragers were categorized as generalists. The proportion of nectar to pollen observations differed between colonies, so the expected values used to calculate specialization did as well. Only bees with at least 15 total foraging observations were included in the specialization categories. Statistics were computed in SPSS 21, except for the exact binomial tests, which were computed in R. Differences between groups (pollen specialists, nectar specialists, generalists, non-foragers, and bees with <15 foraging observations) in ovary size, body size and lipid content were analyzed using a generalized linear model with a gamma distribution and a log link function because these response variables were not normally distributed. Body size was analyzed using a normal distribution and identity link function. All generalized linear models included foraging category and colony as factors and the two non-target variables as covariates. Bivariate correlations were calculated using Spearman’s rank correlations, controlling for colony.

## Results

### Foraging specialization

**Figure 1:**
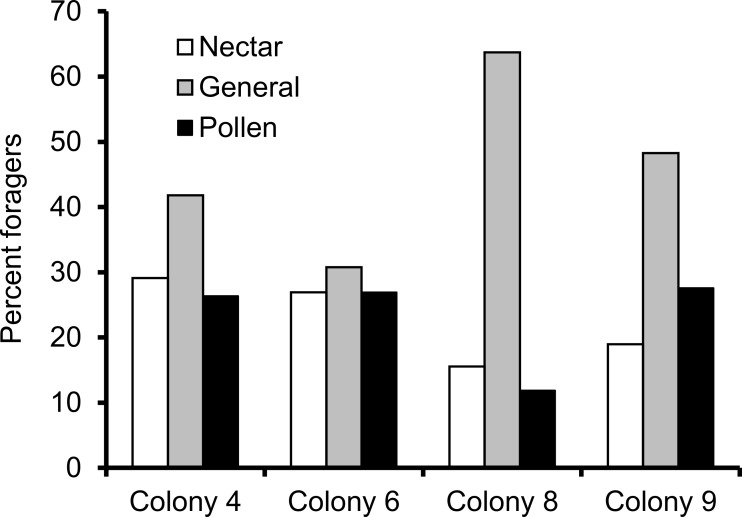
Proportion of foragers in each colony with at least 15 foraging observations that were either nectar specialists (white bars), pollen specialists (black bars) or generalists (gray bars). Proportion of specialists was not uniform across colonies.

**Figure 2:**
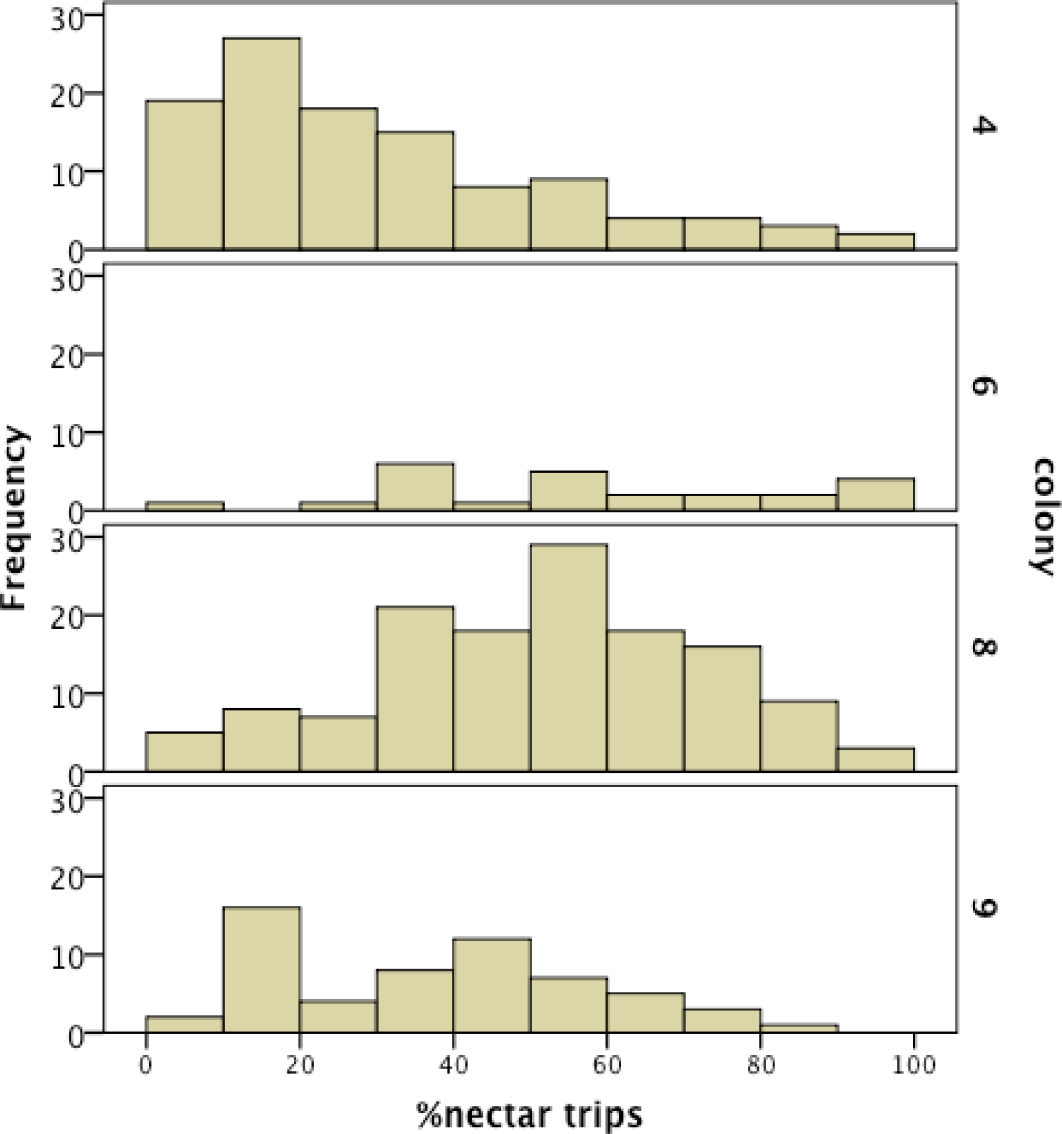
Frequency histogram of the number of individuals (*y* axis) in each colony with a given total percentage of nectar observations. Only individuals with at least 15 foraging observations are included. Each colony contained a mix of specialists and generalists.

We recorded 14,648 visits at our feeders (mean = 25.48 ± 30.76 SD, median = 17, range = 0–202 foraging visits per bee). All four colonies contained both nectar and pollen specialists, as well as individuals that never foraged. Foragers with fewer than 15 observations were not assigned to a foraging or non-foraging category (Table 1). In all colonies, generalists were the most common category, but in but the relative proportions of each group varied between colonies (Fig. 1). All colonies contained bees with a range of foraging preferences, although the distribution was not identical across colonies (Fig. 2). Among those bees with at least 15 trips, the median percent nectar ± interquartile range was 23 ± 29, 52.5 ± 41, 52 ± 27, and 36 ± 35 for colonies 4, 6, 8, and 9, respectively.

**Figure 3:**
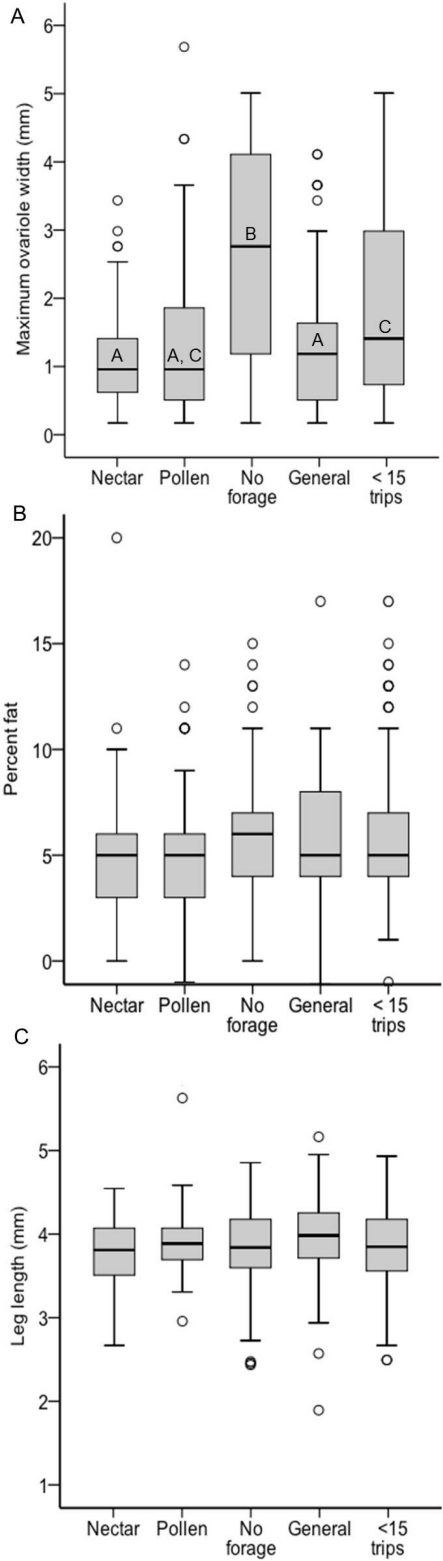
Boxplots of ovary size (A), percent lipid content (B) and body size (C) for each foraging category, pooled across colonies. In (A) bars with different letters significantly differed from each other.

There was a significant effect of foraging category on ovary size across all colonies, but it was driven not by pollen and nectar specialist differences, but by the larger ovaries of non-foragers relative to foragers (*χ*^2^ = 26.37, df = 4, *p* < 0.0001; Fig. 3A). Non-foragers had significantly larger ovaries than other groups (Bonferroni post-hoc comparisons, all *P* < 0.005), and bees with fewer than 15 trips had larger ovaries than pollen and generalist, but not nectar foragers. There were no significant differences in ovary size among the three forager classes (pollen, nectar, generalist; all post-hoc comparisons *P* > 0.05). Body lipid content did not differ significantly between foraging categories (*χ*^2^ = 5.23, df = 4, *p* = 0.27; Fig. 3B). There was no significant effect of foraging category on body size (*χ*^2^ = 13.87, df = 4, *p* = 0.01; Fig. 3C). There was a significant relationship between ovary size and body size, with larger bees having somewhat larger ovaries (*σ* = 0.13, *N* = 483, *p* = 0.006), but no other relationships between ovary size, body size and lipid content (*P* > 0.05 in all cases).

Limiting the analysis to only the foragers (nectar specialists, pollen specialists, and generalists) allows analysis of specialization, i.e., percentage of observations at the nectar feeder, as a continuous variable. However, there were no significant relationships between specialization and ovary size or body size (*p* > 0.05 in all cases, ESM Fig. 1). There was a correlation between percent nectar visits and lipid content (*σ* = 0.13, *N* = 273, *P* = 0.03). Within colony analysis shows that in Colony 8 there was a significant negative correlation between ovary size and specialization, with pollen specialists having larger ovaries (*σ* =  − 0.21, *N* = 125, *P* = 0.02; ESM Fig. 2). However, this relationship was not present in the other three colonies (*p* > 0.05 in all cases).

**Figure 4:**
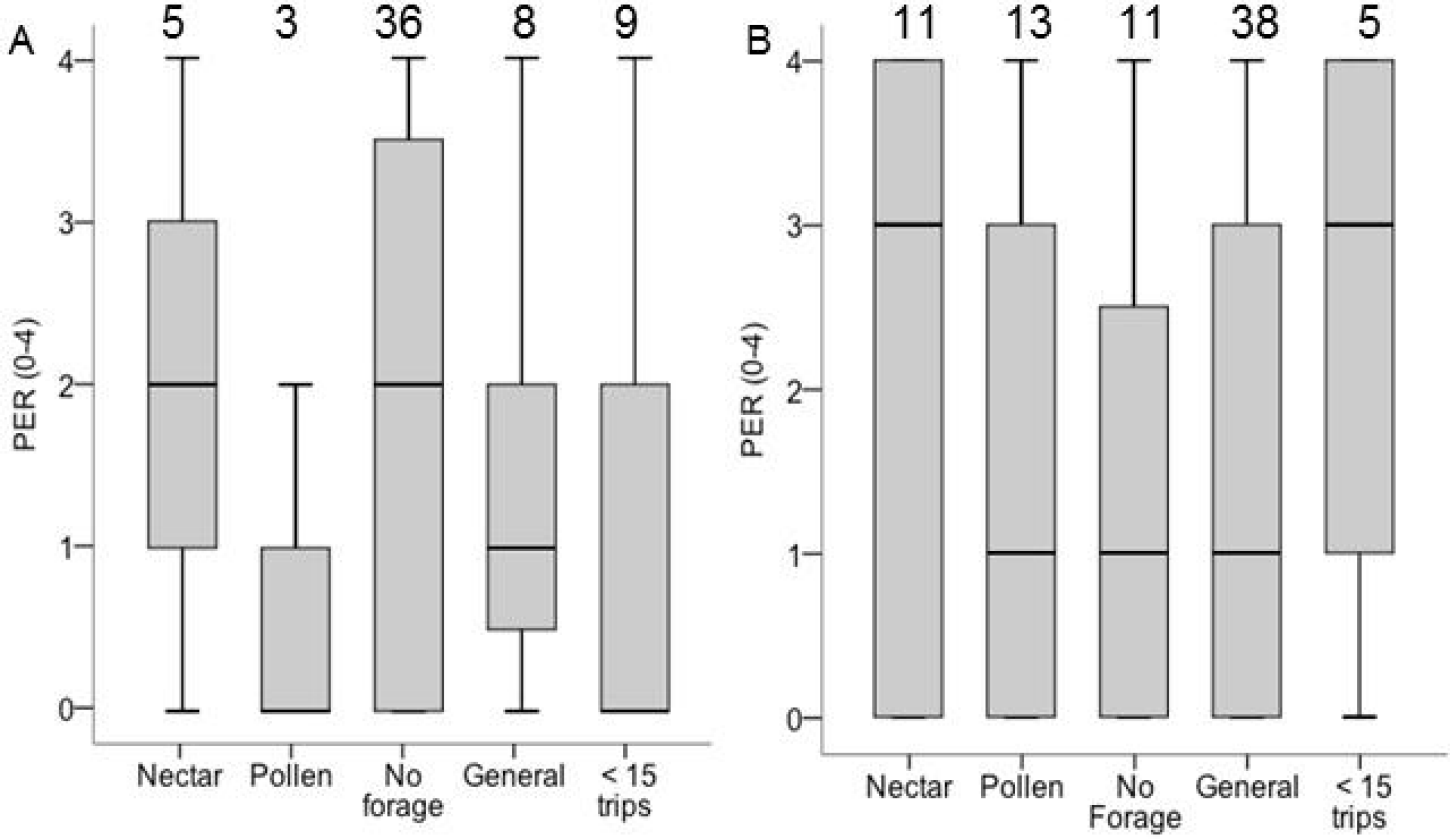
Boxplots PER scores for each foraging category in colony 6 (A) and colony 8 (B). Samples sizes are listed above each bar. There were no significant differences between groups of PER score and ovary size for colony 8 and colony 6.

The analysis of PER and sensory sensitivity included only Colonies 6 and 8. PER score did not differ between groups (*χ*^2^ = 3.32, df = 4, *p* = 0.51; Fig. 4), and in neither colony did specialization correlate with PER score among foragers. In Colony 6, ovary size correlated with PER (*σ* = 0.31, *N* = 59, *P* = 0.02), but not in Colony 8 (*σ* =  − 0.04, *N* = 73, *p* = 0.75; ESM Fig. 3). Pooled across both colonies, among foragers (nectar, pollen, generalist) there was no correlation between PER and ovary size (*σ* =  − 0.04, *N* = 73, *p* = 0.72). Among non-foragers, PER score correlated with ovary size (*σ* = 0.31, *N* = 59, *P* = 0.03). There were no correlations between PER score and lipid content or body size (*p* > 0.05 in all cases).

## Discussion

Here we quantitatively demonstrate pollen and nectar specialization among *B. terrestris* foragers for the first time. Verhaeghe et al. (1999) described variation in pollen and nectar foraging among individuals in free-foraging *B. terestris* colonies, but did not test individual proportions against colony totals. Likewise, Konzmann & Lunau (2014) reported that some bees foraged exclusively for nectar in lab colonies of *B. terrestris*, but did not investigate individual nectar/pollen proportions relative to colony totals. Foraging specialization has been previously demonstrated for *B. bifarius* and *B. impatiens* (O’Donnell, Reichardt & Foster, 2000; Hagbery & Nieh, 2012). Hagbery & Nieh (2012) demonstrated that foragers are responsive to colony needs: specialists adjusted their behavior to compensate for experimentally removed nestmates. The wide variation in median nectar/pollen ratios across the four colonies in our study (Fig. 2) also suggests that individual specialization may be responsive to colony resource needs.

Our data offer only mixed support for the hypothesis that *Bombus* foraging specialization is linked to ovarian activation or differences in sensory sensitivity. The main predictions were not met: there were no ovary size differences between nectar and pollen specialists, nor any differences in sensory sensitivity as expressed through PER. Some results, however, do support predictions derived from the RGPH for honeybees. In Colony 8, bees that specialized most on pollen also had the most developed ovaries. In Colony 6, ovary development correlated with sensory sensitivity. Among non-foragers (that is, those with most developed ovaries) sensory sensitivity also correlated with ovary activation. But overall, we did not find evidence in *B. terrestris* of the striking links between sensory tuning, reproductive physiology, and foraging behavior seen in honeybees (Amdam et al., 2004; Amdam et al., 2006; Page et al., 2006; Page, Rueppell & Amdam, 2012; Page & Amdam, 2007).

The small sample size for PER of Colony 6 foragers likely limited our power to detect potential differences, as did the general lack of responsiveness to the PER assay of bumblebees compared to honeybees. Also, we tested PER across a relatively narrow range of sucrose concentrations, and with a lower sample size than in honeybee studies. PER testing may not be able to distinguish variations in sensory sensitivity in bumblebees as it can in honeybees because bumblebees only show the PER response to high concentrations of sucrose (Laloi et al., 1999; Riveros & Gronenberg, 2009).

However, despite the limited power of the PER data, we show that there was substantial variation in ovary activation and lipid stores between individuals, but neither of these two variables correlated with foraging specialization. Studies of ovary activation and foraging behavior are valuable for testing links between pollen/nectar specialization and reproductive physiology (e.g., Rueppell, Hunggims & Tingek, 2008; Tan et al., 2015), and our results suggest that further investigation of the physiological mechanisms underlying pollen/nectar specialization in bumblebees will provide fruitful comparisons with honeybees.

Currently we have no understanding of the mechanisms underlying foraging specialization in bumblebees. Also, the stingless bees, Meliponini, the sister taxon to bumblebees (Danforth et al., 2013), have pollen/nectar specialist foragers (Sommeijer et al., 1983; Biesmeijer & Tóth, 1998). Further research into the mechanisms of specialization in bumblebees, as well as comparative studies with stingless bees, would provide greater insight into the evolution of specialist foraging. Do all three groups use similar mechanisms, or has the same phenotype evolved through multiple routes? Also, to our knowledge the assumption that solitary bees adjust sensory tuning and foraging preference in concert with ovarian development has never been tested. Understanding solitary bee foraging preferences would illuminate how derived foraging specializations have evolved.

Our data support the “Reproducive Conflict and Work” hypothesis; workers with more developed ovaries avoid foraging effort (Schmid-Hempel, 1990; Roth et al., 2014). Our most striking result was that non-foragers had larger ovaries than any of the forager groups. This is consistent with studies on other *Bombus* species which showed that non-foraging *B. impatiens* workers were more likely than foragers to enlarge their ovaries following queen removal (Jandt & Dornhaus, 2011), or have larger ovaries when the colony naturally entered the competition phase (Foster et al., 2004). In both studies it is not clear if there is a causal relationship between lack of foraging and ovary size, and if so, in which direction. Other studies have found that individuals with larger ovaries forage less in ants (Ito & Higashi, 1991; Powell & Tschinkel, 1999), and wasps (Cant & Field, 2001), suggesting that the general pressure to conserve energy and avoid the mortality associated with foraging in order to pursue reproductive opportunities may be widespread. Given this, we predicted that ovary size would correlate with lipid stores, either because bees with large ovaries avoided foraging, or because those with energy reserves could more readily enlarge their ovaries. However, we found no significant different differences between whole body lipid content and foraging classes (consistent with Couvillon et al., 2011 for *B. impatiens*). This may be due to the controlled laboratory set-up, in which foragers did not engage in the normal, energetically costly foraging flights to collect resources. Additionally, foragers were not exposed to natural predators, which may have influenced bees’ decisions to forage.

Bumblebees are an excellent group with which to study the evolution of foraging specialization. Unlike honeybees, bumblebees have relatively small (∼150 workers), and annual colonies in which colony size and nutrition needs are often in flux as the colony grows and senesces (Heinrich, 1979; Goulson, 2010). Yet, in the three species studied in detail, some foragers in all colonies are resource specialists (O’Donnell, Reichardt & Foster, 2000; Hagbery & Nieh, 2012, this study). Understanding the underlying mechanisms and regulation would shed much light on the potential evolutionary pathways of the more sophisticated specialization exhibited by larger colony social insects.

##  Supplemental Information

10.7717/peerj.2599/supp-1 Data S1 Original data used for all analyses The ”Data” tab of the Excel file contains all data, while the ”Explanation of variable names” explains each column heading in the ”Data” tab. Details of measurement and collection for each variable are in the Methods section of the main text.

10.7717/peerj.2599/supp-2 Supplemental Information 1 Supplementary material

## Additional Information and Declarations

### Data Availability

The following information was supplied regarding data availability: The raw data has been supplied as a Supplementary File.
