# Supplementary material for: Specialization on pollen or nectar in bumblebee foragers is not associated with ovary size, lipid reserves or sensory tuning
Source: PeerJ. 2016 Oct 27;4:e2599. doi: 10.7717/peerj.2599 (PMC5088620; doi:10.7717/peerj.2599)
Supplement: Supplemental Information 1 [file peerj-04-2599-s002.docx]

**Electronic Supplementary Material, Smith et al. Bombus foraging.**


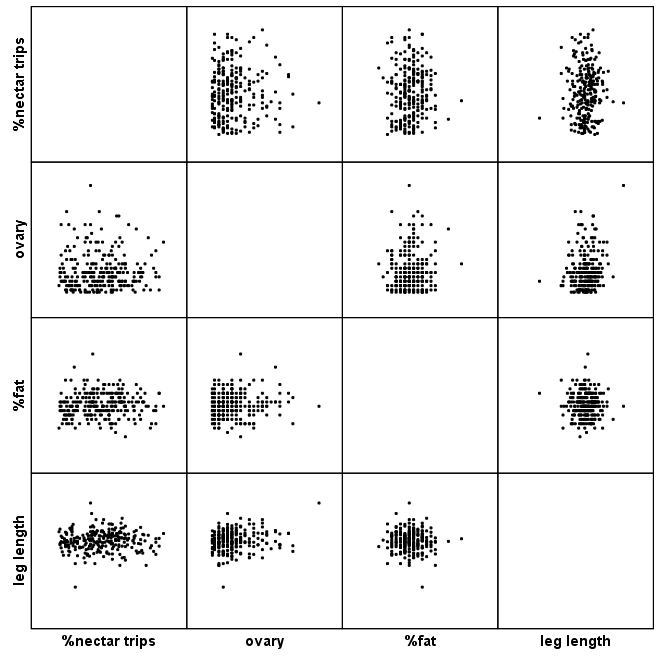


**ESM Figure 1.** Correlations between fat content, ovary size, foraging specialization (measured as percent of all foraging observations on the nectar feeder) and body size (leg length). Only foragers with 15 or more observations are included.


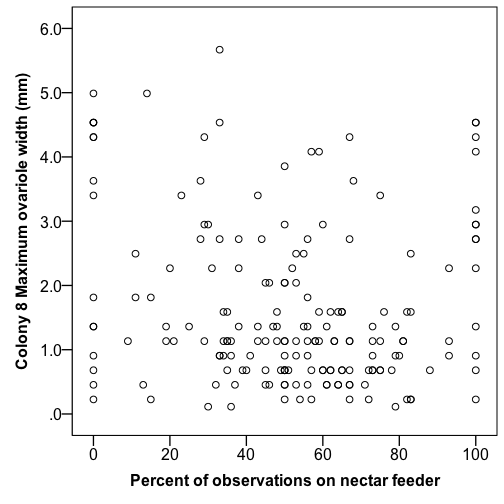


**ESM Figure 2.** Scatterplot of foraging specialization (measured as the percent of all foraging observations on the nectar feeder) and ovary size for colony 8.


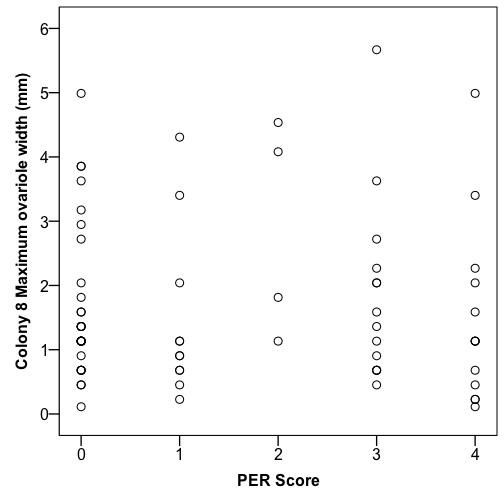

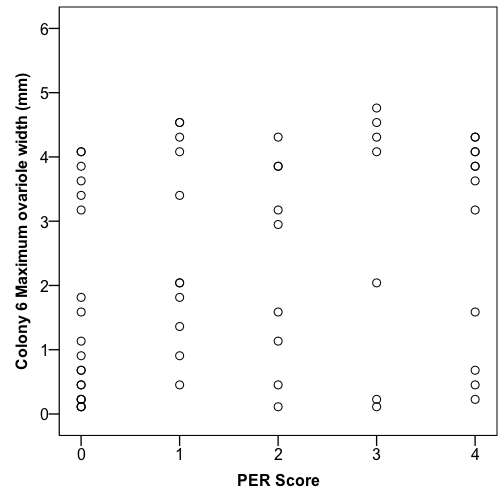


**ESM Figure 3.** Scatterplot
